# Items

**Published:** 1875-03

**Authors:** 


American Medical Association.—The next meeting of the American Medical Association will be held at Louisville, Ky., commencing Tuesday, May 4, 1875. At its annual meeting in January last, the Erie County Medical Society failed to elect delegates to the convention. We notice in the list of delegates from the State Society the names of Drs. J. P. White, T. F. Rochester, C. C. Wyckoff, and J. F. Miner of this city.——Delegate to the Vermont State Medical Society Dr. J. C. Greene of this city, was elected a delegate to the Vermont Medical Society, at the last meeting of the State Society.——Scarlatina. From what we can learn in conversation with physicians, scarlet fever, which has prevailed so extensively in this city is on the decrease.——Alchemy Improved. A writer in the *Ohio Medical and Surgical Reporter* (Homeopathic) says that the alchemic system of medicine has reached "its greatest perfection in the therapeutic principles of Homeopathy."——A Bee Story. We are informed by good authority that an apparently intelligent person in this city lately procured twelve bees and, after macerating them in hot water, drank the fluid as medicine, as the desired result was not obtained it is said to have been for the reason that the bees were not *alive* when the hot water was poured on them. Here seems to be a chance for Mr. Bergh, a little good common sense may not come amiss either.——Appointments. Dr. J. H. Green, of the class of "75," has received the appointment of Senior Resident Physician at the Rochester City Hospital, and Dr. Chas. Cary, of the same class, has been appointed Assistant Physician. Dr. Bernard Bartow has been reappointed Resident Physician to the Buffalo General Hospital.——Small Pox in Montreal. From the *Canada Medical and Surgical Journal* we learn that during the past year there have been 981 deaths from small pox in Montreal. Of these, 827 were among the French-Canadians, and may be looked upon as the direct result of the teachings of a large number of French-Canadian physicians who are opposed to vaccination. The authorities are however not entirely excusable as we understand that no suitable regulations are inforced to insure the isolation of cases of the disease.——Change in the Medical Board of Bellevue Hospital. The New York City Board of Commissioners of Public Charities and Corrections have passed a resolution reinstating those members of the medical and surgical staff who lost their positions in 1874. The resolution takes effect March 15th, after which date the Medical Board will consist of twenty-four members. At the same time resolutions were passed creating a department in the hospital for diseases of women, and one for orthopædic surgery and diseases of children.——Engravings of Distinguished Physicians. Owing to the difficulty experienced in getting the work properly done, the editor of the *Richmond and Louisville Medical Journal* has abandoned his idea of presenting a portrait of some distinguished physician to his subscribers with each number of the Journal. In place of this he has decided to present a large engraving representing Harvey explaining the circulation of the blood.——PERSONAL. Dr. J. P. White, of this city, leaves town in a few days for a trip to California and the Pacific coast. He expects to be accompanied by a party of medical friends from the eastern part of the State, and to be absent about two months. We wish them a pleasant trip and a safe return.

